# Scimitar Syndrome in an Asymptomatic Adult: Fortuitous Diagnosis by Imaging Technique

**DOI:** 10.1155/2012/138541

**Published:** 2012-01-15

**Authors:** Miguel Angel Ramirez-Marrero, Manuel de Mora-Martin

**Affiliations:** Cardiology Department, Regional University Hospital Carlos Haya, Calle Padang 4C, 29190 Malaga, Spain

## Abstract

Congenital cardiopathies in adults are a rare clinical entity in the cardiology consultations. Advances in imaging techniques allow the fortuitous diagnosis of mild forms of these congenital abnormalities. We describe a case of an asymptomatic 41-year-old man, with a medical history of recurrent pneumonia during childhood and an established diagnosis of scimitar syndrome by computed tomography.

## 1. Introduction

Scimitar syndrome is a rare complex congenital anomaly in which the anomalous pulmonary venous return of all or most of the right lung to the inferior vena cava is just below or above the right hemidiaphragm. This malformation is classically divided into infants and adult forms, this last with better prognosis. Traditionally, the initial diagnosis was established by chest X-radiography and echocardiography, being completed by cardiac catheterization. The increasing use of imaging techniques (computed tomography and magnetic resonance imaging) currently leads to increasing diagnosis of asymptomatic forms.

The present paper describes an asymptomatic patient with a fortuitous diagnosis of scimitar syndrome by computed tomography.

## 2. Case Presentation

**Figure 1:**
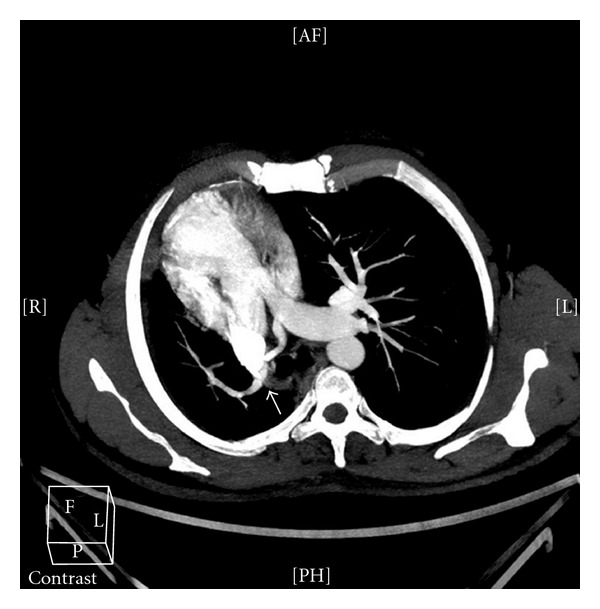
Axial plane showing cardiac dextroposition and hypoplasia of the right lung and right pulmonary artery (white arrow).

**Figure 2:**
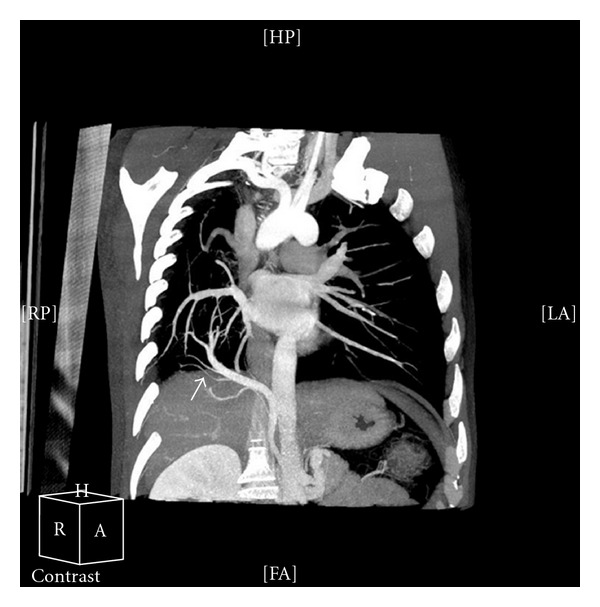
Coronal plane showing the anomalous venous drainage (white arrow).

**Figure 3:**
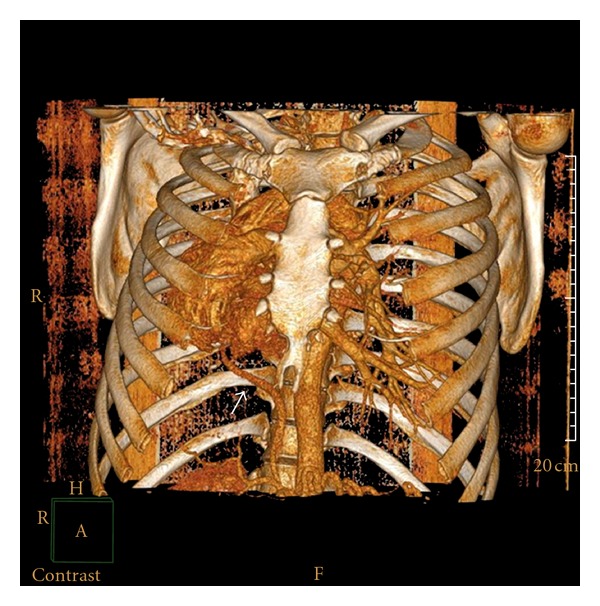
Three-dimensional reconstructed image showing the anomalous venous drainage (white arrow).

**Figure 4:**
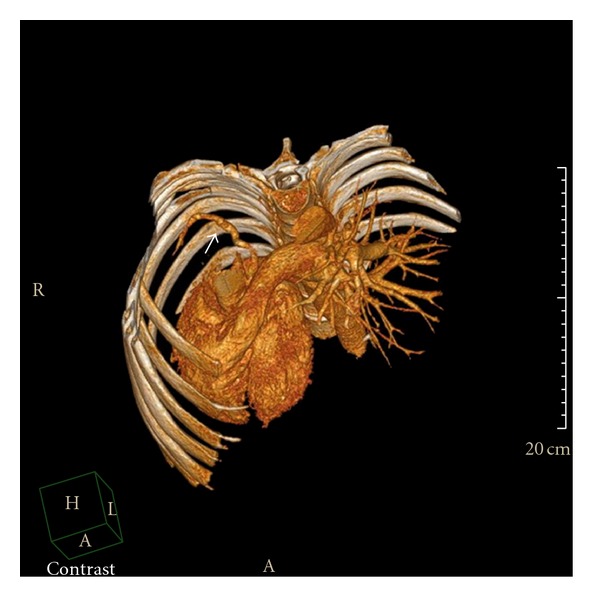
Three-dimensional reconstructed image showing an aortopulmonary collateral artery (white arrow).

A 41-years-old man diagnosed with obstructive sleep apnea-hypopnea syndrome by pneumologist was referred for cardiology consultation for assessment of dextrocardia. Medical history revealed absence of cardiovascular symptoms and recurrent pneumonia with normal growth during childhood. On physical examination, he was in good general condition, lungs were clear, and he had no cardiac murmur. The ECG showed right axis deviation with no signs of right ventricular hypertrophy. Chest X-radiography demonstrated a pronounced dextrocardia. Echocardiographic examination showed cardiac chambers of normal size and contractility, hypoplasia of the right pulmonary artery, and absence of signs of pulmonary hypertension neither intracardiac shunt. Computed tomography demonstrated a cardiac dextroposition, hypoplasia of the right lung and right pulmonary artery (Figure 1), an anomalous venous drainage of the right lung to the subphrenic inferior vena cava (Figures 2 and 3) and aortopulmonary collateral artery (Figure 4), confirming, the diagnosis of scimitar syndrome. It was decided a close monitoring in cardiology consultation, because patient was asymptomatic, without data of pulmonary hypertension. The patient remains asymptomatic at present after completing a 7-year followup.

## 3. Discussion

Scimitar syndrome is a rare complex congenital anomaly (1–3/1,000,000 live births), in which the anomalous pulmonary venous return of all or most of the right lung to the inferior vena cava just below or above the right hemidiaphragm creates the image of a Turkish sword on the chest X-radiography [1]. The mean age of diagnosis is seven months. This malformation is classically divided into infants and adult forms [2]. Infant patients typically develop heart failure due to the significant left-to-right shunt from the anomalous pulmonary venous drainage or from another cardiac defect such as atrial septal defect. Adult patients usually have a benign clinical course; however, they may develop heart failure or repeated respiratory infections. Traditionally, the initial diagnosis in most patients with the scimitar syndrome is established by chest X-radiography and is completed by echocardiography [3]. Cardiac catheterization should always be performed to confirm the diagnosis, identify the course of the anomalous venous drainage, measure the degree of left-to-right shunt, determinate the presence of scimitar vein stenosis and pulmonary hypertension, and detect any associated cardiac abnormalities [4]. The increasing use of imaging techniques (computed tomography and magnetic resonance imaging) leads to increasing diagnosis of asymptomatic forms [5, 6], as the patient described in our case report, is helpful if the scimitar is obscured by the overlying cardiac shadow.

Surgical intervention is indicated for large left-to-right shunt exceeding 50%, resulting in pulmonary hypertension and heart failure, and lung sequestration or recurrent right-sided chest infection [7]. Our patient has no indication of surgical repair and remains currently asymptomatic in scheduled reviews.

## References

[1] Lee M (2007). Isolated and complex scimitar vein anomalies and their differentiation from the meandering right pulmonary vein. *Yonsei Medical Journal*.

[2] Takeda S, Imachi T, Arunitsu K, Minami M, Hayakawa M (1994). Two cases of scimitar variant. *Chest*.

[3] Espinola-Zavaleta N, Játiva-Chávez S, Muñoz-Castellanos L, Zamora-González LMCC (2006). Clinical and echocardiographic characteristics of scimitar syndrome. *Revista Espanola de Cardiologia*.

[4] Brown JW, Ruzmetov M, Minnich DJ (2003). Surgical management of scimitar syndrome: an alternative approach. *Journal of Thoracic and Cardiovascular Surgery*.

[5] Gavazzi E, Ravanelli M, Farina D, Chiari ME, Maroldi R (2008). Scimitar syndrome comprehensive, noninvasive assessment with cardiovascular magnetic resonance imaging. *Circulation*.

[6] Inoue T, Ichihara M, Uchida T, Sakai Y, Hayashi T, Morooka S (2002). Three-dimensional computed tomography showing partial anomalous pulmonary venous connection complicated by the scimitar syndrome. *Circulation*.

[7] Schramel FM, Westermann CJ, Knaepen PJ, Van den Bosch JMM (1995). The scimitar syndrome: clinical spectrum and surgical treatment. *European Respiratory Journal*.

